# Highly frequent promoter methylation and *PIK3CA *amplification in non-small cell lung cancer (NSCLC)

**DOI:** 10.1186/1471-2407-11-147

**Published:** 2011-04-20

**Authors:** Meiju Ji, Haixia Guan, Cuixia Gao, Bingyin Shi, Peng Hou

**Affiliations:** 1 Department of Endocrinology, The First Affiliated Hospital of Xi'an Jiaotong University College of Medicine, Xi'an 710061, the People's Republic of China; 2 Department of Endocrinology and Metabolism, The First Affiliated Hospital of China Medical University, Shenyang 110001, the People's Republic of China

**Keywords:** Promoter methylation, PI3K/Akt pathway, *PIK3CA *amplification, non-small cell lung cancer (NSCLC), clinicopathologic characteristics

## Abstract

**Background:**

Lung cancer is the leading cause of cancer-related death worldwide. Genetic and epigenetic alterations have been identified frequently in lung cancer, such as promoter methylation, gene mutations and genomic amplification. However, the interaction between genetic and epigenetic events and their significance in lung tumorigenesis remains poorly understood.

**Methods:**

We determined the promoter methylation of 6 genes and *PIK3CA *amplification using quantitative methylation-specific PCR (Q-MSP) and real-time quantitative PCR, respectively, and explore the association of promoter methylation with *PIK3CA *amplification in a large cohort of clinically well-characterized non-small cell lung cancer (NSCLC).

**Results:**

Highly frequent promoter methylation was observed in NSCLC. With 100% diagnostic specificity, excellent sensitivity, ranging from 45.8 to 84.1%, was found for each of the 6 genes. The promoter methylation was associated with histologic type. Methylation of *CALCA, CDH1, DAPK1*, and *EVX2 *was more common in squamous cell carcinomas (SCC) compared to adenocarcinomas (ADC). Conversely, there was a trend toward a higher frequency of *RASSF1A *methylation in ADC than SCC. In addition, *PIK3CA *amplification was frequently found in NSCLC, and was associated with certain clinicopathologic features, such as smoking history, histologic type and pleural indentation. Importantly, aberrant promoter methylation of certain genes was significantly associated with *PIK3CA *amplification.

**Conclusions:**

Our data showed highly frequent promoter methylation and *PIK3CA *amplification in Chinese NSCLC population, and first demonstrated the associations of gene methylation with *PIK3CA *amplification, suggesting that these epigenetic events may be a consequence of overactivation of PI3K/Akt pathway.

## Background

Lung cancer is the number one cancer killer in China and soon will reach epidemic levels worldwide [1]. Epidemiological evidence has documented that approximately 41.8 men and 19.3 women per 100,000 Chinese individuals died of lung cancer in 2005 [2]. This disease is largely associated with smoking. While in developed countries smoking rates are decreasing, the use of tobacco products is increasing in the developing countries. In combination with a spike in the number of lung cancer cases in never smokers, this ensures that lung cancer will remain a major health problem [3]. Lung cancer is clinically divided into two subtypes, small cell lung cancer (SCLC) and non-small cell lung cancer (NSCLC). The latter is the most common type, accounting for 85-90% of the total cases [3]. Although recent studies have shown that adjuvant chemotherapy improves survival in completely resected NSCLC [4,5], only 5-15% of treated individuals ultimately benefit [6]. Despite the fact that the cause of most lung cancer is well know, the disease has proven difficult to diagnosis early and treat successfully, reflecting limited advances in our understanding of the molecular mechanisms underlying lung carcinogenesis.

Currently, mutations in the *K-ras *oncogene, *p53 *tumor suppressor gene, and epidermal growth factor receptor (*EGFR*) gene have been found frequently in lung tumors and implicated in lung carcinogenesis [7-9]. In addition, genomic abnormalities represent another major signature of neoplastic transformation and tumor progression [10]. Chromosome copy number abnormalities in lung cancer have been frequently identified using comparative genomic hybridization (CGH) assay [11,12], including genomic amplification of *PIK3CA *which codes the phosphatidylinositol-3-kinase (PI3K) catalytic subunit α [13]. PIK3CA is generally activated by a series of cell surface tyrosine kinase receptors [14]. Upon activation, PIK3CA binds to its heterodimer, p85, and promotes the phosphorylation of Akt. Activated Akt phosphorylates down-stream protein effectors and amplifies the signaling cascade, enhancing cell proliferation and survival [15]. The previous studies have shown that *PIK3CA *amplification was more frequently observed in squamous cell carcinomas (SCC) than adenocarcinomas (ADC), which was closely associated with increased Akt activity in SCC [12,13], suggesting that *PIK3CA *amplification, in addition to *K-ras *and *EGFR *mutations, may be another major cause of overactivation of PI3K/Akt pathway that promotes lung tumorigenesis.

In addition to genetic factors, promoter methylation is an alternative mechanism underlying inactivation of tumor-associated genes in lung carcinogenesis [16,17]. Currently, methylated gene profiles have been widely studied in lung cancer [18,19]. Of note, although it has been suggested that epigenetic alterations of genes can occur as a consequence of, or coexist with, aberrant signaling of certain oncogenic pathways activated by genetic alterations, such as *PIK3CA *amplification, *EGFR *mutation and *K-ras *mutation [20,21], the interaction between genetic and epigenetic alterations in lung cancer still remains poorly understood.

In the present study, we used quantitative methylation-specific PCR (Q-MSP) to evaluate methylation levels of a panel of cancer-related genes in a cohort of clinically well-characterized NSCLC samples, including *CALCA, CDH1, DAPK1, EVX2, PAX6*, and *RASSF1A*, and further explore the association of promoter methylation of these genes with *PIK3CA *amplification.

## Methods

### Clinical samples and DNA isolation

With the institutional review board approval, 96 tissue samples from 96 NSCLC patients and 15 controls from non-cancerous respiratory diseases, including 6 patients with pulmonary tuberculosis, 4 patients with brochiectasis and 5 patients with pulmonary abscess, were randomly obtained from the First Affiliated Hospital of China Medical University, P.R. China. Of these controls, the mean age was 60.7 years, and over 60 years of age accounts for 47%. Sixty-seven percent of controls were male. Seventy-three percent of controls had a history of smoking. The clinicopathologic characteristics of NSCLC cases are shown in Table 1. None of these NSCLC patients received chemotherapy and radiotherapy before the surgery. Informed consent was obtained from each NSCLC patient before the surgery. All of the samples were reviewed by a pathologist at Department of Pathology of the Hospital to identify histologic type and other tumor characteristics. The samples were treated and genomic DNA was isolated from paraffin-embedded tissues as previously described [22], using xylene to remove pareffin and sodium dodecyl sulfate (SDS) and proteinase K to digest tissues, followed by phenol-chloroform extraction and ethanol precipitation of DNA.

**Table 1:** Clinicopathologic features of NSCLC cases

Characteristics	No. of patients (%)
Gender	
Male	66 (69)
Female	30 (31)
	
Age (mean years ± S.D.)	58.9 ± 9.2
≤ 60	56 (58)
> 60	40 (42)
	
Smoking history	
No	30 (31)
Yes	66 (69)
	
Tumor size (mean cm ± S.D.)	3.9 ± 1.7
1-3	37 (38)
3-5	44 (46)
> 5	15 (16)
	
Histologic type	
Adenocarcinoma (ADC)	30 (31)
Squamous (SSC)	66 (69)
	
Histologic stage	
I	54 (56)
II	32 (34)
III	10 (10)
	
Lymph node metastasis	
No	70 (73)
Yes	26 (27)
	
Pleural indentation	
No	75 (78)
Yes	21 (22)
	
Invasion or Adhesion	
No	65 (68)
Yes	31 (32)

### Sodium bisulfite treatment

Bisulfite processing of DNA was performed in principle as described previously [20]. Briefly, a final volume of 20 μl of H_2_O containing 2 μg genomic DNA, 10 μg salmon sperm DNA, and 0.3 M NaOH was incubated at 50°C for 20 min to denature the DNA. The bisulfite reaction was performed in 500 μl of a freshly prepared solution containing 3 M sodium bisulfite (Sigma, Saint Louis, MO), 10 mM hydroquinone (Sigma, Saint Louis, MO) at 70°C for 2-3 h. DNA was subsequently recovered by a Wizard DNA Clean-Up System (Promega Corp., Madison, WI) following the instructions of the manufacturer, and desulphonated in 0.2 M NaOH at 37°C for 10 minutes, neutralized by ammonium acetate, alcohol precipitated, dried, and then dissolved in 30 μl of deionized H_2_O. After bisulfite processing, all unmethylated cytosine residues converted to uracil, whereas the methylated cytosine residues remained unchanged. Bisulfited-modified DNA samples were stored at -80°C until use.

### Quantitative methylation-specific PCR (Q-MSP) assay

The Q-MSP protocol was as described previously [20]. Briefly, the Q-MSP assay was carried out in a final volume of 20 μl on a 96-well plate using an ABI 7500 Fast Real-Time PCR System (Perkin-Elmer, Foster City, CA). The reaction mixture contained 3 μl bisulfite-treated DNA, 600 nM each primer, 200 nM TaqMan probe, 5.5 mM MgCl2, 0.6 U platinum *Taq *polymerase, 200 μM each of deoxyguanosine triphosphate, and 2% Rox reference. Q-MSP was run at 95°C for 2 min, followed by 40 cylces at 95°C for 15 sec and 60°C for 1 min. Each sample was run in triplicate and each plate contained multiple water blanks and serial dilutions of positive methylated controls to construct the standard curve. Methylated DNA samples, used as positive controls, were obtained by *in vitro *treatment of leukocyte DNA with Sss I DNA methylase (New Engliand Biolabs, Beverly, MA). To determine the relative level of methylation, the ratio of the value of the gene of interest over the value of the internal reference gene (*β-actin*) was used in the current detection system. The primers and TaqMan probes used in the present study were presented in Table 2.

**Table 2:** Quantitative methylation-specific PCR primer and TaqMan probe sequences used in the present study

Genes	Forward primer sequence (5'→3')	Probe sequence (5'→3')	Reverse primer sequence (5'→3')
***CALCA***	GTTTTGGAAGTATGAGGGTGACG	6FAM-ATTCCGCCAATACACAACAACCAATAAACG-TAMRA	TTCCCGCCGCTATAAATCG

***CDH1***	AATTTTAGGTTAGAGGGTTATCGCGT	6FAM-CGCCCACCCGACCTCGCAT-TAMRA	TCCCCAAAACGAAACTAACGAC

***DAPK1***	GGATAGTCGGATCGAGTTAACGTC	6FAM-TTCGGTAATTCGTAGCGGTAGGGTTTGG-TAMRA	CCCTCCCAAACGCCGA

***EVX2***	TCGTTGGCGGGTGGGTATAG	6FAM-CTTCACTCCAAACCGCTCCTCATCTCCCG-TAMRA	ACGCCGATAACAACCATTTTAACG

***PAX6***	ATATAGGACGGCGGTTTAGGTTG	6FAM-CCCAAAATCCGACCGACTCCAACCCCTA-TAMRA	TTCCGACCGAACGAAAACCTAC

***RASSF1A***	GCGTTGAAGTCGGGGTTC	6FAM-ACAAACGCGAACCGAACGAAACCA-TAMRA	CCCGTACTTCGCTAACTTTAAACG

***β-Actin***	TGGTGATGGAGGAGGTTTAGTAAGT	6FAM-ACCACCACCCAACACACAATAACAAACACA-TAMRA	AACCAATAAAACCTACTCCTCCCTTAA

### Copy number analysis of *PIK3CA *with real-time quantitative PCR

We used a real-time quantitative PCR technique to analyze the copy number of *PIK3CA *gene on an ABI 7500 Fast Real-Time PCR System (Perkin-Elmer, Foster City, CA) as described previously [22]. This method was well established and validated by florescence *in situ *hybridization (FISH) [22,23], which has been widely used in the various cancers [22-25]. Specific primers and probes were designed using Primer Express 3.0 (Applied Biosystems) to amplify *PIK3CA *and *β-actin *genes as described previously [22]. The reaction of quantitative PCR was repeated twice, and *β-actin *was run in parallel to normalize the amount of input DNA. A standard curve was established using serial dilutions of normal leukocyte DNA with a quantity range of 0.01-20 ng/μl. Amplification of *PIK3CA *gene was defined by a copy number ≥ 4.

### Statistical analysis

A methylation positive result was defined when the ratio was above a certain cut-off value. The relative level of methylation varied significantly among the 6 genes and the cut-off points were thus studied for each gene individually. To define cut-off value of each gene, we construct receiver operating characteristic (ROC) curves using the Medcalc Software (MedCalc Software bvba, Belgium). The area under ROC curve is a measure of the ability of a continuous marker to accurately classify tumor and non-tumor tissue. Such a curve is a plot of sensitivity *vs*. 100 minus specificity values associated with all dichotomous markers that can be formed by varying the cut-off values used to define a marker "positive". As an useful biomarker for early diagnosis and prognostic evaluation of diseases, its specificity should be more important than its sensitivity. Hence, in the present study, cut-offs were obtained in order to achieve 100% specificity. Factors (promoter methylation and *PIK3CA *amplification) associated with clinicopathological characteristics of tumor were assessed univariately using the Medcalc Software (MedCalc Software bvba, Belgium). Similarly, univariate models were examined the association between gene methylation and *PIK3CA *amplification. Multivariate models were then developed that adjusted for the most important covariates, including smoking history, histologic type, and lymph node metastasis. Sample means were compared using unpaired *t*-test, assuming unequal variances, and all tests were two-tailed. *P *values <0.05 were considered significant. All statistical analyses were performed using the SPSS statistical package (11.5, Chicago, IL, USA).

## Results

### NSCLC patient profiles

In the present study, we analyzed the promoter methylation of 6 genes using Q-MSP technique in 96 well-characterized NSCLC patients. As shown in Table 1, the mean age of all NSCLC cases was 58.9 years, and the cases with age >60 years accounts for 42%. Males were more frequent than females (69% *vs*. 31%). Sixty-nine percent of cases had a history of smoking. Ninety percent of NSCLC cases had surgical stage I and II disease and 84% had tumors < 5 cm. By histology, 31% of patients were ADC, and 69% were SCC. The cases with lymph node metastasis, pleural indentation and invasion or adhesion were in 26/96 (27%), 21/96 (22%), and 31/96 (32%), respectively.

### Frequent promoter methylation and *PIK3CA *amplification in NSCLC

**Figure 1:**
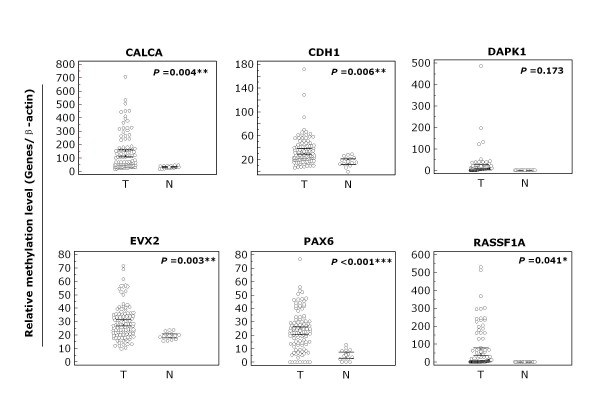
**The overall methylation levels of the 6 genes in NSCLC**. Q-MSP assay was performed as described in Methods. The relative methylation level (on *Y *axis) is represented by ratios of candidate gene/*β-actin *(*CALCA, CDH1, DAPK1*, and *RASSF1A *× 1000; *EVX2 *and *PAX6 *× 100). Horizonal lines indicate a 95% confidence interval for the sample mean. T, tumor tissues; N, non-cancerous lung tissues. *, *P *< 0.05; **, *P *< 0.01; ***, *P *< 0.001.

**Figure 2:**
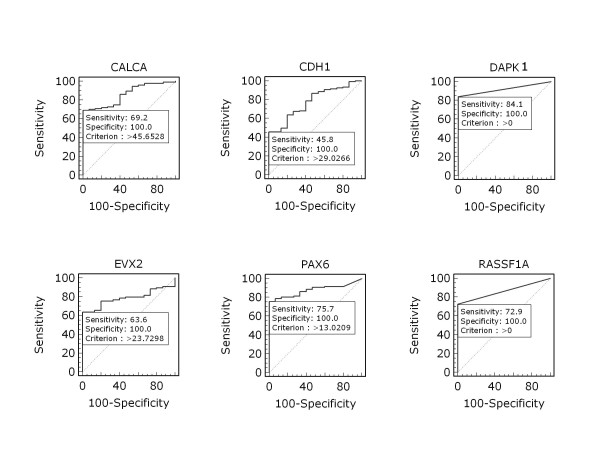
**Receiver operating characteristic (ROC) curves for the 6 genes in NSCLC**. Complete DNA methylation data from all NSCLC and non-cancerous lung tissues were used to construct the ROC curves. The ROC curves plot sensitivity *vs*. 100-specificity. The determined cut-off values for *CALCA, CDH1, DAPK1, EVX2, PAX6*, and *RASSF1A *were 45.7, 29.0, 0, 23.7, 13.0, and 0, respectively.

We used Q-MSP assay to examine promoter methylation of *CALCA, CDH1, DAPK1, EVX2, PAX6*, and *RASSF1A *genes in a large cohort of NSCLC samples. As shown in Figure 1, the overall methylation level of each of 6 genes was higher in NSCLC tissues than in non-cancerous lung tissues, particularly *CALCA *(*P *< 0.01), *CDH1 *(*P *< 0.01), *EVX2 *(*P *< 0.01), *PAX6 *(*P *< 0.001), and *RASSF1A *(*P *< 0.05). To distinguish NSCLC from non-cancerous lung tissues, we set up appropriate cut-off values, and used them to determine diagnostic sensitivity and specificity. As shown in Figure 2, with 100% diagnostic specificity for each of the 6 genes, the sensitivity of *CALCA, CDH1, DAPK1, EVX2, PAX6*, and *RASSF1A *was 69.2%, 45.8%, 84.1%, 63.6%, 75.7%, and 72.9%, respectively. With a gene copy number of four or more defined as amplification, we found the incidence of *PIK3CA *amplification in NSCLC was 31.3% (30/96) in the present study. Of note, among all cases with *PIK3CA *amplification, only two cases were ADC, the remaining cases were SCC.

### Association of promoter methylaton and *PIK3CA *amplification with clinicopathological characteristics in NSCLC

**Figure 3:**
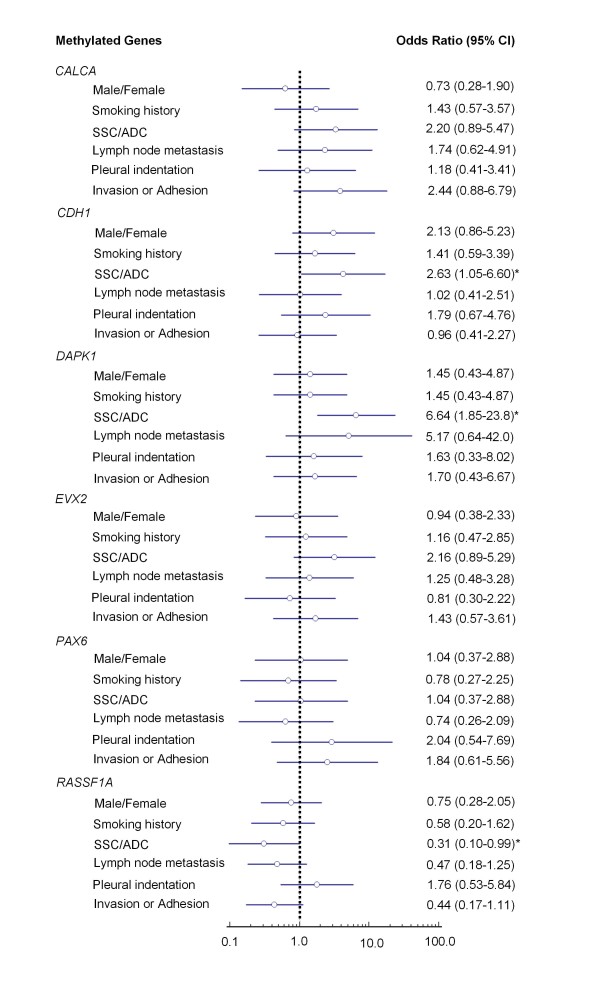
**Relationship of promoter methylation (Odds Ratios) with tumor characteristics of NSCLC**. Multiple univariate logistic-regressions were performed with the use of methylation data for the 6 genes that were defined as 'positive' or 'negative' through receiver operating characteristic (ROC) curves. Details are as described in Methods. *, *P *< 0.05.

Among all clinicopathologic characteristics, promoter methylation was associated with histologic type (Figure 3). The univariate analyses showed that methylation of *CALCA, CDH1, DAPK1*, and *EVX2 *was common in SCC compared to ADC, particularly in *CDH1 *(OR = 2.63, 95% CI = 1.05-6.60) and *DAPK1 *(OR = 6.64, 95% CI = 1.85-23.8) (Figure 3). Conversely, there was a trend toward a higer frequency of *RASSF1A *methylation in ADC than SCC (Figure 3). Moreover, there was a trend toward a positive association between *CALCA *methylation and invasion or adhesion (OR = 2.44, 95% CI = 0.88-6.79). Similarly, there was a trend toward a higher frequency of *DAPK1 *methylaton in patients with lymph node metastasis than without metastasis (OR = 5.17, 95% CI = 0.64-42.0) (Figure 3). Of interest, *RASSF1A *methylation was negatively associated with smoking history, lymph node metastasis, and invasion or adhesion, respectively, although these associations did not reach statistical difference (Figure 3), suggesting that *RASSF1A *methylation may be an early event in lung tumorigenesis, which further confirmed a previous study. However, no association was found between promoter methylation and other clinocopathologic characteristics, including gender, age, quantity of cigarette smoking, tumor size, and pleural indentation (data not shown).

**Figure 4:**
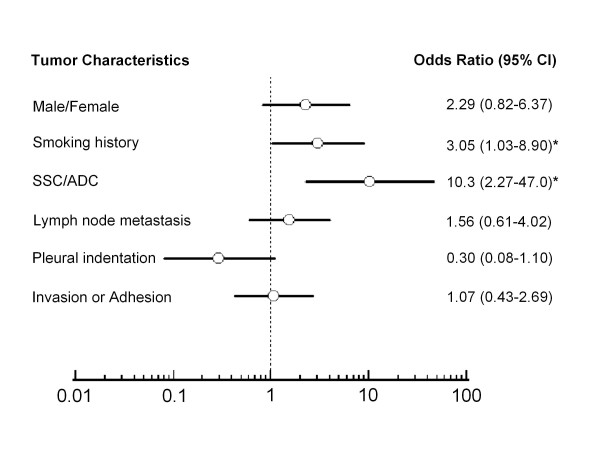
**Relationship of *PIK3CA *amplification (Odds Ratios) with tumor characteristics of NSCLC**. Univariate logistic regression was performed to evaluate the relationship between *PIK3CA *amplification and clinicopathological characteristics of NSCLC cases. Details are as described in Methods. *, *P *< 0.05.

Similar to a previous study [25], *PIK3CA *gene was more frequently amplified in NSCLC, and was not amplified in non-cancerous lung tissues in the present study. The results showed that there was a trend toward a higher frequency of *PIK3CA *amplification in males than females (24/66 in males and 6/30 in females) (Figure 4). *PIK3CA *amplification was significantly frequent in smokers compared to never-smokers (25/66 in smokers and 5/30 in never-smokers; OR = 3.05, 95% CI = 1.03-8.90), and in SCC compared to in ADC (28/66 in SCC and 2/30 in ADC; OR = 10.3, 95% CI = 2.27-47.0) (Figure 4). Of note, there was a trend toward a negative association between *PIK3CA *amplification and pleural indentation in NSCLC (Figure 4).

### Association of promoter methylation with *PIK3CA *amplification in NSCLC

**Figure 5:**
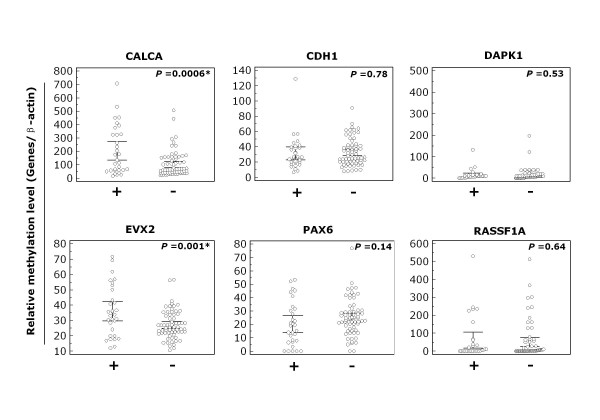
**Association of promoter methylation of these 6 genes with *PIK3CA *amplification in NSCLC**. Methylation levels of the 6 genes are represented as described in Figure 1. '+' indicates tumor samples harbor *PIK3CA *amplification; '-' indicates lack of *PIK3CA *amplification. *, *P *< 0.05.

To explore the relationship between promoter methylation with *PIK3CA *amplification in NSCLC, all tumor samples were divided into two groups, one with *PIK3CA *amplification and the other without *PIK3CA *amplification. As shown in Figure 5, methylation level of *CALCA *(*P *= 0.0006) and *EVX2 *(*P *= 0.001) was significantly higher in the former group than in the latter group. However, there was not significant association between *PIK3CA *amplification and methylation level of other genes, including *CDH1, DAPK1, PAX6*, and *RASSF1A *(Figure 5), suggesting a specific association of *PIK3CA *amplification with methylation of *CALCA *and *EVX2 *in NSCLC.

Univariate analyses indicated that promoter methylation of *CALCA *was significantly positively associated with *PIK3CA *amplification (OR = 3.05, 95% CI = 1.03-8.99). Conversely, promoter methylation of *PAX6 *was significantly negatively associated with *PIK3CA *amplification (OR = 0.27, 95% CI = 0.10-0.72). In order to assess the independent associations between promoter methylation of these genes and *PIK3CA *amplification, smoking history, histologic type, and lymph node metastasis, we conducted multiple multivariable logistic regressions (Table 3). Similarly, the results showed that there was a trend toward a positive association between *CALCA *methylation and *PIK3CA *amplification (OR = 2.55, 95% CI = 0.81-8.08). Promoter methylation of *PAX6 *was significantly negatively associated with *PIK3CA *amplification (OR = 0.19, 95% CI = 0.06-0.62) (Table 3).

**Table 3:** Promoter methylation of individual genes in NSCLC - multivariable models assessing *PIK3CA *amplification, smoking history, histologic type, and lymph node metastasis (OR^† ^and 95%CI)

Genes	*PIK3CA *amplification	Smoking history	SSC *vs*. ADC	Lymph node metastasis
***CALCA***	2.55 (0.81-8.08)	1.08 (0.41-2.84)	1.46 (0.51-4.16)	1.38 (0.44-4.31)

***CDH1***	0.42 (0.16-1.12)	1.35 (0.52-3.47)	4.01 (1.37-11.79)*	0.64 (0.24-1.75)

***DAPK1***	0.59 (0.11-3.15)	1.45 (0.39-5.30)	6.09 (1.25-29.66)*	2.26 (0.24-21.44)

***EVX2***	1.31 (0.47-3.69)	0.94 (0.36-2.43)	2.06 (0.73-5.84)	0.92 (0.32-2.65)

***PAX6***	0.19 (0.06-0.62)*	0.97 (0.31-3.11)	2.62 (0.68-10.14)	0.62 (0.19-2.05)

***RASSF1A***	1.63 (0.57-4.70)	0.65 (0.22-1.95)	0.32 (0.09-1.18)	0.67 (0.24-1.87)

† OR: odds ratio with 95% confidence interval, adjusted for multiple comparisions.

* Significant at *P *< 0.05.

## Discussion

In the present study, we investigated the promoter methylation of the 6 genes in a large cohort of well-characterized NSCLC samples using Q-MSP technique and their relationships to *PIK3CA *amplification. Promoter methylation of these genes, as a mechanism for their silencing, has been frequently observed in NSCLC, particularly *CALCA, CDH1, DAPK1*, and *RASSF1A *[26-28]. In addition, this study also involved two new methylation markers, *EVX2 *and *PAX6*, which were highly specific for tumor-associated methylation, and little or no methylation was observed in tumor-adjacent normal lung tissue [29]. Our findings showed that the overall methylation level from 5 of 6 genes in tumor tissues was significant higher than in non-cancerous lung tissues, including *CALCA, CDH1, EVX2, PAX6*, and *RASSF1A*. With 100% diagnostic specificity, excellent sensitivity, ranging from 45.8 to 84.1%, was obtained for each of the 6 genes. However, the analysis of hypermethylation still had a limitation in the present study, which the values of hypermethylation were not established a priori, but were calculated to maximize sensitivity given 100% specificity, the resulting sensitivities might be biased overestimates. To obtain non-biased sensitivity (and specificity) values, cut-offs need to be validated in a separate independent sample. In the present study, we also observed that promoter methylation of certain genes was associated with histologic type. Methylation of *CALCA, CDH1, DAPK1*, and *EVX2 *was common in SCC compared to ADC, particularly in *CDH1 *(OR = 2.63, 95% CI = 1.05-6.60) and *DAPK1 *(OR = 6.64, 95% CI = 1.85-23.8). Conversely, there was a trend toward a higher frequency of *RASSF1A *methylation in ADC than SCC, which is consistent with a recent study [26]. Of note, there was a trend toward an association between methylation of *CALCA *and *DAPK1 *and invasion or adhesion and lymph node metastasis, suggesting that aberrant methylation of these genes is associated with oncologic outcomes of NSCLC patients. Similar to a previous study [30], *RASSF1A *methylation was negatively associated with smoking history, lymph node metastasis, and invasion or adhesion, respectively. In addition, *RASSF1A *was more frequently methylated in early tumor stage (data not shown), suggesting that *RASSF1A *methylation may be an early event in lung tumorigenesis.

Although tobacco smoking plays a dominant role in the development of lung cancer, we did not observe significant association between promoter methylation and smoking history in the present study, in agreement with most studies [31-34]. However, several studies have reported aberrant methylation of tumor-related genes was associated with tobacco smoking [32,35,36]. It is possible that smoking-associated lung cancer is complex disease which involved many unique genetic and epigenetic events. Thus, better understanding of the molecular mechanisms underlying this disease would undoubtedly improve the outcomes of such patients.

It has recently become clear that PI3K/Akt pathway is frequently activated in human cancers [15,37], and plays an important role in the regulation of cell growth, proliferation, and survival and is involved in human tumorigenesis [15]. In recent years, many oncologists have mainly focused on PIK3CA. In addition to *PIK3CA *mutations [38,39], *PIK3CA *amplification was frequently found in lung cancer, and promoted lung tumorigenesis through overactivation of PI3K/Akt signaling pathway [12,13,25,39]. Similar to the previous studies, in the present study, we found high frequency of *PIK3CA *amplification in Chinese NSCLC population. Moreover, *PIK3CA *amplification was significantly associated with smoking history and histologic type, which was more frequent in smokers compared to never-smokers, and in SCC compared to in ADC. Of note, although no statistical difference was noted, *PIK3CA *amplification was negatively associated with pleural indentation in NSCLC. However, pleural indentation is a well-known imaging sign on chest computed tomography (CT) that suggests a possible pleural invasion by peripheral NSCLC, particularly ADC [40,41]. Importantly, pleural involvement was significantly correlated with a poor prognosis in NSCLC, suggesting that pleural involvement may be one of most important factors to affect on the prognosis of NSCLC [42]. One possibility to explain this contradiction is that, *PIK3CA *gene was more frequently amplified in SCC, not ADC, whereas pleural indentation is more common in ADC.

It has been suggested the epigenetic alterations might addict cells to certain oncogenic pathways, predisposing cells to the accumulations of genetic mutations, which drives tumor progression [43]. On the other hand, overactivation of certain oncogenic pathways can affect the activity of methytransferase, and potentially the methylation activity and regulation of gene transcription, such as RAS/RAF/MEK/ERK pathway (MAPK pathway) [44-47]. Recently, a number of tumor-related genes were found to be aberrantly methylated in association with the MAPK pathway overactivated by *BRAF *mutation in human cancers, such as *hMLH1 *in colon cancer [48], and *SLC5A8 *in thyroid cancer [49]. Of note, our previous study showed that *PTEN *gene was aberrantly methylated in association with activating genetic alterations in PI3K/Akt pathway, including *PIK3CA *amplification [20]. Moreover, another study showed that the differences in the evolvement of epigenetic alterations between the *EGFR *and *K-ras *mutation-mediated tumorigenesis and suggested that the specific interation of genetic and epigenetic events in lung tumorigenesis [21]. In the present study, we found that promoter methylation of *CALCA, EVX2*, and *PAX6 *was significantly associated with *PIK3CA *amplification in NSCLC, however, such association was not seen with other gene methylation, suggesting that epigenetic alterations of these three genes may specifically occur as a consequence of overactivation of PI3K/Akt pathway in NSCLC.

## Conclusions

In summary, the results of the present study provided evidence that multiple genes were aberrantly methylated during the process of lung tumorigenesis. We found highly frequent *PIK3CA *amplification in Chinese NSCLC cases, but not in non-cancerous lung tissues, implicating PI3K/Akt pathway in lung tumorigenesis. Importantly, we have the first time revealed significant associations of gene methylation with *PIK3CA *amplification in NSCLC, which is consistent with a model which aberrant methylation and hence silencing of a number of tumor-related genes, which coexisted with activating genetic alterations of PI3K/Akt pathway, may be a consequence of overactivation of this pathway. Further studies are needed to explore the molecular mechanisms underling the link between such genetic and epigenetic events.

## Competing interests

The authors declare that they have no competing interests.

## Authors' contributions

MJ and PH conceived and designed the experiments. MJ, HG and CG performed the experiments. MJ and PH collected the samples and analyzed the data. BS and PH contributed reagents/materials/analysis tools. MJ and PH Wrote the paper. All authors are in agreement with the content of the manuscript and this submission.

## Pre-publication history

The pre-publication history for this paper can be accessed here:

http://www.biomedcentral.com/1471-2407/11/147/prepub

## References

[B1] Song F He M Li H Qian B Wei Q Zhang W Chen K Hao X A cancer incidence survey in Tianjin: the third largest city in China-between 1981 and 2000 Cancer Causes Control 2008 19 443 450 10.1007/s10552-007-9105-6 18095173

[B2] Yang L Parkin DM Li LD Chen YD Bray F Estimation and projection of the national profile of cancer mortality in China: 1991-2005 Br J Cancer 2004 90 2157 2166 15150609 10.1038/sj.bjc.6601813 PMC2409509

[B3] Molina JR Yang P Cassivi SD Schild SE Adjei AA Non-small cell slung cancer: epidemiology, risk factors, treatment, and survivorship Mayo Clin Proc 2008 83 584 594 10.4065/83.5.584 18452692 PMC2718421

[B4] Kato H Ichinose Y Ohta M Hata E Tsubota N Tada H Watanabe Y Wada H Tsuboi M Hamajima N Japan Lung Cancer Research Group on Postsurgical Adjuvant Chemotherapy. A randomized trial of adjuvant chemotherapy with uracil-tegafur for adenocarcinoma of the lung N Engl J Med 2004 350 1713 1721 10.1056/NEJMoa032792 15102997

[B5] Winton T Livingston R Johnson D Rigas J Johnston M Butts C Cormier Y Goss G Inculet R Vallieres E National Cancer Institute of Canada Clinical Trials Group; National Cancer Institute of the United States Intergroup JBR.10 Trial Investigators. Vinorelbine plus cisplatin vs. observation in resected non-small-cell lung cancer N Engl J Med 2005 352 2589 2597 10.1056/NEJMoa043623 15972865

[B6] Hotta K Matsuo K Ueoka H Kiura K Tabata M Tanimoto M Role of adjuvant chemotherapy in patients with resected non-small-cell lung cancer: reappraisal with a meta-analysis of randomized controlled trials J Clin Oncol 2004 22 3860 3867 10.1200/JCO.2004.01.153 15326194

[B7] Keohavong P DeMichele MA Melacrinos AC Landreneau RJ Weyant RJ Siegfried JM Detection of K-ras mutations in lung carcinomas: relationship to prognosis Clin Cancer Res 1996 2 411 418 9816185

[B8] Le Calvez F Mukeria A Hunt JD Kelm O Hung RJ Tanière P Brennan P Boffetta P Zaridze DG Hainaut P TP53 and KRAS mutation load and types in lung cancers in relation to tobacco smoke: distinct patterns in never, former, and current smokers Cancer Res 2005 65 5076 5083 10.1158/0008-5472.CAN-05-0551 15958551

[B9] Kosaka T Yatabe Y Endoh H Kuwano H Takahashi T Mitsudomi T Mutations of the epidermal growth factor receptor gene in lung cancer: biological and clinical implications Cancer Res 2004 64 8919 8923 10.1158/0008-5472.CAN-04-2818 15604253

[B10] Gray JW Collins C Genome changes and gene expression in human solid tumors Carcinogenesis 2000 21 443 452 10.1093/carcin/21.3.443 10688864

[B11] Balsara BR Sonoda G du Manoir S Siegfried JM Gabrielson E Testa JR Comparative genomic hybridization analysis detects frequent, often high-level, overrepresentation of DNA sequences at 3q, 5p, 7p, and 8q in human non-small cell lung carcinomas Cancer Res 1997 57 2116 2120 9187106

[B12] Bjorkqvist AM Husgafvel-Pursiainen K Anttila S Karjalainen A Tammilehto L Mattson K Vainio H Knuutila S DNA gains in 3q occur frequently in squamous cell carcinoma of the lung, but not in adenocarcinoma Genes Chromosomes Cancer 1998 22 79 82 10.1002/(SICI)1098-2264(199805)22:1<79::AID-GCC11>3.0.CO;2-D 9591638

[B13] Massion PP Kuo WL Stokoe D Olshen AB Treseler PA Chin K Chen C Polikoff D Jain AN Pinkel D Genomic copy number analysis of non-small cell lung cancer using array comparative genomic hybridization: implications of the phosphatidylinositol 3-kinase pathway Cancer Res 2002 62 3636 3640 12097266

[B14] Vanhaesebroeck B Stein RC Waterfield MD The study of phosphoinositide 3-kinase function Cancer Surv 1996 27 249 270 8909804

[B15] Vivanco I Sawyers CL The phosphatidylinositol 3-Kinase AKT pathway in human cancer Nat Rev Cancer 2002 2 489 501 10.1038/nrc839 12094235

[B16] Palmisano WA Divine KK Saccomanno G Gilliland FD Baylin SB Herman JG Belinsky SA Predicting lung cancer by detecting aberrant promoter methylation in sputum Cancer Res 2000 60 5954 5958 11085511

[B17] Risch A Plass C Lung cancer epigenetics and genetics Int J Cancer 2008 123 1 7 10.1002/ijc.23605 18425819

[B18] Zochbauer-Muller S Fong KM Virmani AK Geradts J Gazdar AF Minna JD Aberrant promoter methylation of multiple genes in non-small cell lung cancers Cancer Res 2001 61 249 255 11196170

[B19] Safar AM Spencer H Su X Coffey M Cooney CA Ratnasinghe LD Hutchins LF Fan CY Methylation profiling of archived non-small cell lung cancer: a promising prognostic system Clin Cancer Res 2005 11 4400 4405 10.1158/1078-0432.CCR-04-2378 15958624

[B20] Hou P Ji M Xing M Association of PTEN gene methylation with genetic alterations in the phosphatidylinositol 3-kinase/AKT signaling pathway in thyroid tumors Cancer 2008 113 2440 2447 10.1002/cncr.23869 18831514

[B21] Toyooka S Tokumo M Shigematsu H Matsuo K Asano H Tomii K Ichihara S Suzuki M Aoe M Date H Mutational and epigenetic evidence for independent pathways for lung adenocarcinomas arising in smokers and never smokers Cancer Res 2006 66 1371 1375 10.1158/0008-5472.CAN-05-2625 16452191

[B22] Wu G Mambo E Guo Z Hu S Huang X Gollin SM Trink B Ladenson PW Sidransky D Xing M Uncommon mutation, but common amplifications, of the PIK3CA gene in thyroid tumors J Clin Endocrinol Metab 2005 90 4688 4693 10.1210/jc.2004-2281 15928251

[B23] Engelman JA Zejnullahu K Mitsudomi T Song Y Hyland C Park JO Lindeman N Gale CM Zhao X Christensen J MET amplification leads to gefitinib resistance in lung cancer by activating ERBB3 signaling Science 2007 316 1039 1043 10.1126/science.1141478 17463250

[B24] Hou P Liu D Shan Y Hu S Studeman K Condouris S Wang Y Trink A El-Naggar AK Tallini G Genetic alterations and their relationship in the phosphatidylinositol 3-kinase/Akt pathway in thyroid cancer Clin Cancer Res 2007 13 1161 1170 10.1158/1078-0432.CCR-06-1125 17317825

[B25] Kawano O Sasaki H Okuda K Yukiue H Yokoyama T Yano M Fujii Y PIK3CA gene amplification in Japanese non-small cell lung cancer Lung Cancer 2007 58 159 160 10.1016/j.lungcan.2007.06.020 17681398

[B26] Hawes SE Stern JE Feng Q Wiens LW Rasey JS Lu H Kiviat NB Vesselle H DNA hypermethylation of tumors from non-small cell lung cancer (NSCLC) patients is associated with gender and histologic type Lung Cancer 2010 69 172 179 10.1016/j.lungcan.2009.11.002 19945765 PMC2888601

[B27] Wang Y Zhang D Zheng W Luo J Bai Y Lu Z Multiple gene methylation of nonsmall cell lung cancers evaluated with 3-dimensional microarray Cancer 2008 112 1325 1336 10.1002/cncr.23312 18286531

[B28] Brock MV Hooker CM Ota-Machida E Han Y Guo M Ames S Glöckner S Piantadosi S Gabrielson E Pridham G DNA methylation markers and early recurrence in stage I lung cancer N Engl J Med 2008 358 1118 1128 10.1056/NEJMoa0706550 18337602

[B29] Rauch TA Zhong X Wu X Wang M Kernstine KH Wang Z Riggs AD Pfeifer GP High-resolution mapping of DNA hypermethylation and hypomethylation in lung cancer Proc Natl Acad Sci USA 2008 105 252 257 10.1073/pnas.0710735105 18162535 PMC2224196

[B30] Honorio S Agathanggelou A Schuermann M Pankow W Viacava P Maher ER Latif F Detection of RASSF1A aberrant promoter hypermethylation in sputum from chronic smokers and ductal carcinoma in situ from breast cancer patients Oncogene 2003 22 147 150 10.1038/sj.onc.1206057 12527916

[B31] Brabender J Usadel H Danenberg KD Metzger R Schneider PM Lord RV Wickramasinghe K Lum CE Park J Salonga D Adenomatous polyposis coli gene promoter hypermethylation in non-small cell lung cancer is associated with survival Oncogene 2001 20 3528 3532 10.1038/sj.onc.1204455 11429699

[B32] Kim DH Nelson HH Wiencke JK Zheng S Christiani DC Wain JC Mark EJ Kelsey KT p16(INK4a) and histology-specific methylation of CpG islands by exposure to tobacco smoke in non-small cell lung cancer Cancer Res 2001 61 3419 3424 11309302

[B33] Kim DH Nelson HH Wiencke JK Christiani DC Wain JC Mark EJ Kelsey KT Promoter methylation of DAP-kinase: association with advanced stage in non-small cell lung cancer Oncogene 2001 20 1765 1770 10.1038/sj.onc.1204302 11313923

[B34] Sanchez-Cespedes M Decker PA Doffek KM Esteller M Westra WH Alawi EA Herman JG Demeure MJ Sidransky D Ahrendt SA Increased loss of chromosome 9p21 but not p16 inactivation in primary non-small cell lung cancer from smokers Cancer Res 2001 61 2092 2096 11280771

[B35] Kersting M Friedl C Kraus A Behn M Pankow W Schuermann M Differential frequencies of p16(INK4a) promoter hypermethylation, p53 mutation, and K-ras mutation in exfoliative material mark the development of lung cancer in symptomatic chronic smokers J Clin Oncol 2000 18 3221 3229 10986054 10.1200/JCO.2000.18.18.3221

[B36] Toyooka S Maruyama R Toyooka KO McLerran D Feng Z Fukuyama Y Virmani AK Zochbauer-Muller S Tsukuda K Sugio K Smoke exposure, histologic type and geography-related differences in the methylation profiles of non-small cell lung cancer Int J Cancer 2003 103 153 160 10.1002/ijc.10787 12455028

[B37] Samuels Y Ericson K Oncogenic PI3K and its role in cancer Curr Opin Oncol 2006 18 77 82 10.1097/01.cco.0000198021.99347.b9 16357568

[B38] Kawano O Sasaki H Endo K Suzuki E Haneda H Yukiue H Kobayashi Y Yano M Fujii Y PIK3CA mutation status in Japanese lung cancer patients Lung Cancer 2006 54 209 215 10.1016/j.lungcan.2006.07.006 16930767

[B39] Okudela K Suzuki M Kageyama S Bunai T Nagura K Igarashi H Takamochi K Suzuki K Yamada T Niwa H PIK3CA mutation and amplification in human lung cancer Pathol Int 2007 57 664 671 10.1111/j.1440-1827.2007.02155.x 17803655

[B40] Shapiro R Wilson GL Yesner R Shuman H A useful roentgen sign in the diagnosis of localized bronchioloalveolar carcinoma Am J Roentgenol Radium Ther Nucl Med 1972 114 516 524 4334808 10.2214/ajr.114.3.516

[B41] Kuhlman JE Fishman EK Kuhajda FP Meziane MM Khouri NF Zerhouni EA Siegelman SS Solitary bronchioloalveolar carcinoma: CT criteria Radiology 1988 167 379 382 2833764 10.1148/radiology.167.2.2833764

[B42] Li M Ito H Wada H Tanaka F Pit-fall sign on computed tomography predicts pleural involvement and poor prognosis in non-small cell lung cancer Lung Cance 2004 46 349 355 10.1016/j.lungcan.2004.05.017 15541820

[B43] Baylin SB Ohm JE Epigenetic gene silencing in cancer - a mechanism for early oncogenic pathway addiction? Nat Rev Cancer 2006 6 107 116 10.1038/nrc1799 16491070

[B44] MacLeod AR Rouleau J Szyf M Regulation of DNA methylation by the Ras signaling pathway J Biol Chem 1995 270 11327 11337 10.1074/jbc.270.19.11327 7744770

[B45] Oelke K Richardson B Decreased T cell ERK pathway signaling may contribute to the development of lupus through effects on DNA methylation and gene expression Int Rev Immunol 2004 23 315 331 10.1080/08830180490452567 15204091

[B46] Pruitt K Ulkü AS Frantz K Rojas RJ Muniz-Medina VM Rangnekar VM Der CJ Shields JM Ras-mediated loss of the pro-apoptotic response protein Par-4 is mediated by DNA hypermethylation through Raf-independent and Raf-dependent signaling cascades in epithelial cells J Biol Chem 2005 280 23363 23370 10.1074/jbc.M503083200 15831492

[B47] Lu R Wang X Chen ZF Sun DF Tian XQ Fang JY Inhibition of the extracellular signal-regulated kinase/mitogen-activated protein kinase pathway decreases DNA methylation in colon cancer cells J Biol Chem 2007 282 12249 12259 17307743 10.1074/jbc.M608525200

[B48] Weisenberger DJ Siegmund KD Campan M Young J Long TI Faasse MA Kang GH Widschwendter M Weener D Buchanan D CpG island methylator phenotype underlies sporadic microsatellite instability and is tightly associated with BRAF mutation in colorectal cancer Nat Genet 2006 38 787 793 10.1038/ng1834 16804544

[B49] Porra V Ferraro-Peyret C Durand C Selmi-Ruby S Giroud H Berger-Dutrieux N Decaussin M Peix JL Bournaud C Orgiazzi J Silencing of the tumor suppressor gene SLC5A8 is associated with BRAF mutations in classical papillary thyroid carcinomas J Clin Endocrinol Metab 2005 90 3028 3035 10.1210/jc.2004-1394 15687339

